# Machine learning approaches for estimation of the fraction of absorbed photosynthetically active radiation and net photosynthesis rate of maize using multi-spectral sensor

**DOI:** 10.1016/j.heliyon.2024.e34117

**Published:** 2024-07-04

**Authors:** Pradosh Kumar Parida, Somasundaram Eagan, Krishnan Ramanujam, Radhamani Sengodan, Sivakumar Uthandi, Parameswari Ettiyagounder, Raja Rajagounder

**Affiliations:** a Department of Agronomy, Tamil Nadu Agricultural University, Coimbatore, 641003, Tamil Nadu, India; b Directorate of Agribusiness Development (DABD), Tamil Nadu Agricultural University, Coimbatore, 641003, Tamil Nadu, India; c Nammazhvar Organic Farming Research Centre, Tamil Nadu Agricultural University, Coimbatore, 641003, Tamil Nadu, India; d Department of Agricultural Microbiology, Tamil Nadu Agricultural University, Coimbatore, 641003, Tamil Nadu, India; e ICAR-Central Institute for Cotton Research (CICR) Regional Station, Coimbatore, 641003, Tamil Nadu, India

**Keywords:** Precision agriculture, Multispectral images, Machine learning, UAV, Vegetation indices

## Abstract

The fraction of absorbed photosynthetically active radiation (FAPAR) and the photosynthesis rate (Pn) of maize canopies were identified as essential photosynthetic parameters for accurately estimating vegetation growth and productivity using multispectral vegetation indices (VIs). Despite their importance, few studies have compared the effectiveness of multispectral imagery and various machine learning techniques in estimating these photosynthetic traits under high vegetation coverage. In this study, seventeen multispectral VIs and four machine learning (ML) algorithms were utilized to determine the most suitable model for estimating maize FAPAR and Pn during the *kharif* and *rabi* seasons at Tamil Nadu Agricultural University, Coimbatore, India. Results demonstrate that indices such as OSAVI, SAVI, EVI-2, and MSAVI-2 during the *kharif* and MNDVIRE and MSRRE during the *rabi* season outperformed others in estimating FAPAR and Pn values. Among the four ML methods of random forest (RF), extreme gradient boosting (XGBoost), support vector regression (SVR), and multiple linear regression (MLR) considered, RF consistently showed the most effective fitting effect and XGBoost demonstrated the least fitting accuracy for FAPAR and Pn estimation. However, SVR with R^2^ = 0.873 and RMSE = 0.045 during the *kharif* and MLR with R^2^ = 0.838 and RMSE = 0.053 during the *rabi* season demonstrated higher fitting accuracy, particularly notable for FAPAR prediction. Similarly, in the prediction of Pn, MLR showed higher fitting accuracy with R^2^ = 0.741 and RMSE = 2.531 during the *kharif* and R^2^ = 0.955 and RMSE = 1.070 during the *rabi* season. This study demonstrated the potential of combining UAV-derived VIs with ML to develop accurate FAPAR and Pn prediction models, overcoming VI saturation in dense vegetation. It underscores the importance of optimizing these models to improve the accuracy of maize vegetation assessments during various growing seasons.

## Highlights

• Proposed a new method to predict Pn from FAPAR using multispectral VIs.
• OSAVI & MNDVIRE were the most accurate for maize photosynthetic traits estimation.
• Various machine learning algorithms were applied and compared.
• RF achieved the best fine-tuning; SVR & MLR had the highest accuracy in predictions.

## Graphical abstract

**Figure:**
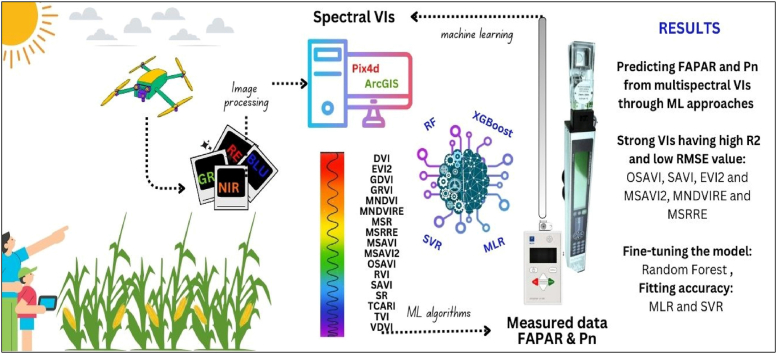

## 1 Introduction

Green vegetation utilizes sunlight to produce food through photosynthesis, a crucial process for its overall function. Photosynthetically active radiation (PAR) is the solar radiation that fuels this activity within the range of 400–700 nm. Fraction of absorbed photosynthetically active radiation (FAPAR) is the proportion of solar energy that enters this spectrum and is absorbed by the canopy of plants. In ecosystems, FAPAR is an essential measure of the carbon balance, and energy distribution, as well as determining the productivity and health of plants [1,2]. FAPAR is also essential for ecosystem models, plant productivity models, agricultural yield estimation models, and climate models for monitoring environmental changes including drought and the effects of climate change by understanding the physiology of vegetation [[3], [4], [5], [6]]. Improving these modelling efforts requires effective and precise techniques for mapping FAPAR over wide regions [7]. Net photosynthesis, which takes into account losses from respiration and plant mortality, concentrates on the actual increase of dry matter, whereas the rate of photosynthesis is a general measure of this process. It's more useful and significant information to understand the overall growth and production of a plant. FAPAR is directly proportional to the net photosynthetic rate (Pn), indicating the primary productivity of photosynthesis in crops and ecosystems [8].

The fraction of absorbed photosynthetically active radiation (FAPAR) can be measured by different methods, including radiation sensors like SunScan and AccuPAR, conventional ground-based methods such as canopy analysis systems, and Pn can be measured with sensors like infrared gas analyser and portable photosynthesis system. Although these techniques measure accurate and up-to-date FAPAR and Pn data, they are excessively time-consuming, labour-intensive, and unsuitable for extensive evaluations [[9], [10], [11]]. In contrast, although remote sensing provides a non-destructive method of obtaining spectral information from plant canopies, it overcomes the drawbacks of ground-based measurements [12,13]. Among the VIs, enhanced vegetation index (EVI), Green normalized difference vegetation index (GNDVI), normalized difference vegetation index (NDVI), perpendicular vegetation index (PVI), and simple ratio (SR), have been widely utilized to estimate field-measured FAPAR using UAV based multispectral remote sensing [14]. Multispectral remote sensing may provide a rapid estimation of FAPAR and Pn across large areas, still in the case of densely vegetated areas, the saturation effect in VIs causes challenges. A recent study has shown that indices that use the red edge (RE) bands between 680 and 780 nm are useful for estimating a variety of plant biophysical characteristics [15,16]. Furthermore, compared to other bands like NDVI, the RE band has been employed more frequently to measure photosynthetic vegetation (PV) [17].

Unmanned aerial vehicles (UAVs) equipped with multi-sensors offer significant advantages in ecological and environmental research due to their easy deployment, flexibility in flying altitude and data acquisition dates, and high temporal and spatial resolutions [[18], [19], [20], [21]]. They are increasingly replacing satellite platforms in various applications, including vegetation modelling, mapping, and monitoring. Their use extends to predicting FAPAR values, water stress, canopy temperature, and agricultural yields [[22], [23], [24], [25]]. The high volume of data obtained, such as high spatial-temporal resolution multi-spectral images, have great potential in optimizing agronomic practices to increase crop yields and quality in precision agriculture [[26], [27], [28]].

The algorithm of choosing is one of the aspects that affect the estimation accuracy of FAPAR. These techniques, which include logarithmic, linear, exponential, and quadratic polynomial models as examples, use VIs to estimate FAPAR. Various collinearities between VIs and FAPAR or, other biophysical parameters may develop as crops mature and VIs get closer to saturation because of robust foliage. Machine learning methods are being used more and more to address this issue, including Extreme Gradient Boosting (XGBoost), Random Forest (RF), and Support Vector Regression (SVR). Studies have found that SVR can provide better accuracy than artificial neural networks (ANN) [29], while RF and XGBoost compared to other machine learning models, studies have demonstrated that they have the potential to reduce overfitting [[30], [31], [32], [33]]. It is still necessary to carefully accessed the accuracy of RF, XGBoost, SVR, and MLR in particular for Pn estimate. Furthermore, a limited number of studies investigated the effect of various VI combinations on FAPAR estimates, highlighting areas that require further research to enhance FAPAR and Pn prediction through machine learning methods.

Therefore, the objectives of this study were to 1) to investigate the potential ability of multispectral-based VIs for estimating maize photosynthetic characteristics like FAPAR values of maize during the *kharif* and *rabi* seasons, 2) to evaluate the performance of various ML methods, *viz*., RF, XGB, SVR and MLR in predicting maize FAPAR values, and 3) to test the effectiveness of selected optimal VIs and ML approaches for predicting maize Pn.

## 2 Materials and methods

### 2.1 Location of the study area and studied field

**Fig. 1:**
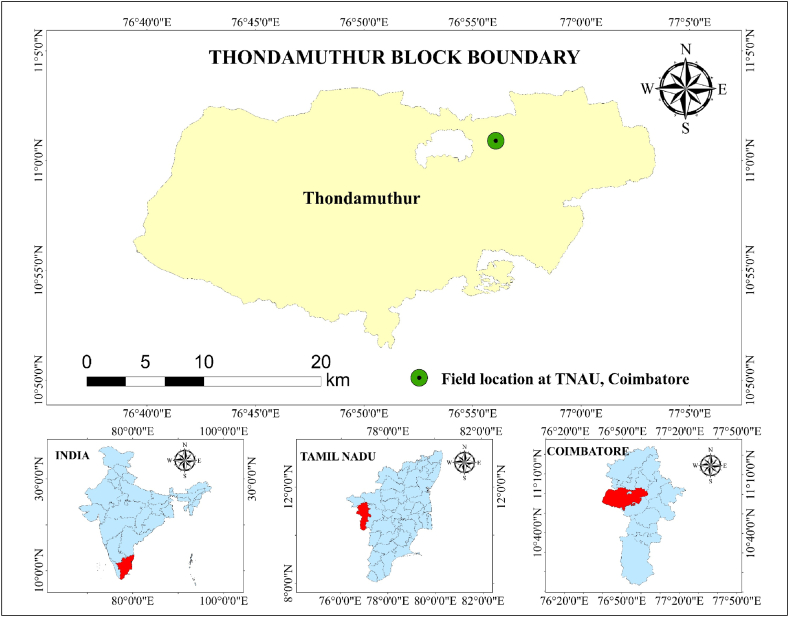
Location of the experimental farm.

The experiment was conducted during the *kharif* and *rabi* seasons of 2023-24 on the maize field of the Eastern Block farm at Tamil Nadu Agricultural University, Coimbatore. The experimental farm's location is shown in Fig. 1. It is located in Tamil Nadu's Western Agroclimatic zone with coordinates of 11°N latitude and 77°E longitude, with an elevation of 426.7 m above mean sea level.

**Fig. 2:**
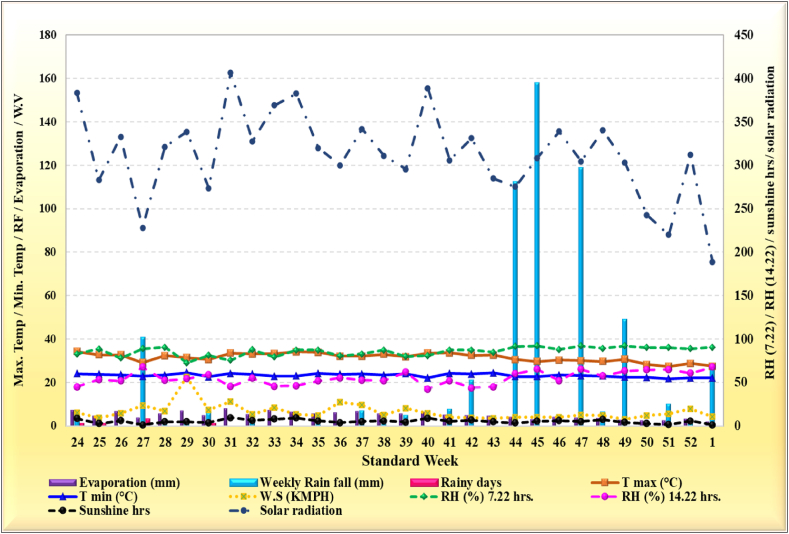
Variation in weather parameters during crop growing period.

Meteorological data were collected from the Department of Agroclimate Research Centre, TNAU, Coimbatore, for the experimental period (Fig. 2). The mean maximum and minimum temperatures of 34.3 °C and 21.7 °C, respectively, were recorded during the 24th and 51st standard meteorological weeks. Throughout the experimentation period, a total rainfall of 5.81 cm was received, with November alone accounting for 67.36 % of this total. Notably, the maximum weekly rainfall of 158 mm was observed during the 45th standard meteorological week. The mean maximum and minimum relative humidity values were 92.10 % and 44.80 %, respectively, recorded during the 45th and 24th standard meteorological weeks. Soil samples were randomly collected from the top 0–30 cm at the experimental site and thoroughly mixed to form a composite sample. Physical and chemical analyses revealed that the soil at the experimental site was classified as a sandy clay loam texture, with an alkaline pH of 8.27, high salinity (0.17 dS m^−1^), and a medium level of organic carbon (4.68 g kg^−1^). The soil exhibited low levels of available nitrogen (238 kg ha^−1^) and phosphorus (22.97 kg ha^−1^) but was high in available potassium (602.43 kg ha^−1^).

### 2.2 Data collection

#### 2.2.1 Image acquisition

A DJI-Phantom 4 multispectral imaging system (Table 1) was mounted to a quadcopter drone for this investigation. The primary advantage of this device is its capacity for vertical take-off and landing, which makes it useful in restricted space. This flight will also be automated using waypoints. Using the ground control station software DJI Ground Station Pro (DJI GS Pro 2.0), the flight mission (waypoints, altitude, heading direction, and speed) was planned. A clear sky aircraft operation was conducted between 11 a.m. and 12 noon to collect multispectral images. The georeferencing process (GCP) made use of a ground reference point. The multispectral camera captures the pictures, which are later downloaded.

**Table 1:** Description of flight information and UAV sensor specifications.

Platform	:	Quadcopter		
Flight speed	:	4 m s^−1^		
Altitude	:	30 m		
Overlap rate	:	75 %		
Ground Sample Distance (GSD)	:	1.52 cm per pixel (per band)		
Multispectral sensor	:	DJI- Phantom 4		
Image spectral band	:	Blue, Green, Red, Red edge, Near IR		
Characteristic wavelength	:	Name	Central wavelength (nm)	Wavelength range (nm)
		Blue (B)	450	±16
		Green (G)	560	±16
		Red (R)	650	±16
		Red edge (RE)	730	±16
		Near IR (NIR)	840	±26
Image resolution (pixels)	:	1600 × 1300		

For radiometric calibration, a calibrated reflectance panel (CRP) was placed on the ground within the survey area, ensuring it was clean and unobstructed. Before each flight, images of the CRP were captured following the panel-specific calibration manual instructions, with the UAV camera positioned to fully frame and illuminate the panel. These pre-flight images were later used to normalize the reflectance values in the multispectral data. After the flight, the CRP images were utilized to adjust the reflectance values of the collected data, compensating for variability in lighting conditions and ensuring consistency across all images [34]. The software applied corrections based on recorded atmospheric conditions, including light intensity and atmospheric composition, ensuring that the reflectance values in the images accurately represented ground conditions. This process mitigated the effects of atmospheric particles and variable light conditions, which could otherwise distort the reflectance values.

#### 2.2.2 Ground-based data collection

The tasselling and silking stages of maize cultivation are the most crucial phases for data collection. Ground truth measurements were obtained for various parameters, including fraction of absorbed photosynthetically active radiation and net photosynthesis rate. Simultaneously, drone images were employed to validate the vegetation indices. To ensure accuracy, ten sets of data were collected at each plot for the validation of the VIs.

*Canopy FAPAR Measurements*: Measurements of the fraction of absorbed photosynthetically active radiation (FAPAR) in maize canopies were made using the Decagon Devices Inc. LP-80 PAR/LAI Ceptometer after UAV images were taken. The LP-80 probe measured PAR in the 400–700 nm range (μmol. m^−2^. s^−1^) using 80 separate sensors arranged at 1 cm intervals. To collect comprehensive PAR data, the probe was placed in four different places and directions: incident PAR, top of canopy reflected PAR, bottom canopy transmission PAR, and soil reflection PAR. Every plot was subjected to five iterations to ascertain the mean value, which was then utilized to compute the maize canopy FPAR using Equation (1).

(1) $$\text{FAPAR}=\frac{\left({PAR}_{ad}-\;{PAR}_{au}\right)-\left({PAR}_{bd}-\;{PAR}_{bu}\right)}{{PAR}_{ad}}\;\ldots$$

Where, above-canopy upwelling PAR (PAR au and downwelling PAR (PAR ad), and below-canopy upwelling PAR (PAR bu) and downwelling PAR (PAR bd)

*Net Photosynthesis Rate (Pn)*: The CI-340 Hand-Held Photosynthesis System measured photosynthetic activity in both controlled environments and the field, being operated with one hand. It involved placing a top third leaf in the CI-340 chamber to obtain a net photosynthesis rate and expressed as *μmol. m*^*−2*^*. s*^*−1*^. Accuracy was ensured through averaging readings, and five points were randomly selected to validate the vegetation indices.

### 2.3 Data processing

#### 2.3.1 Image Mosaicking

**Fig. 3:**
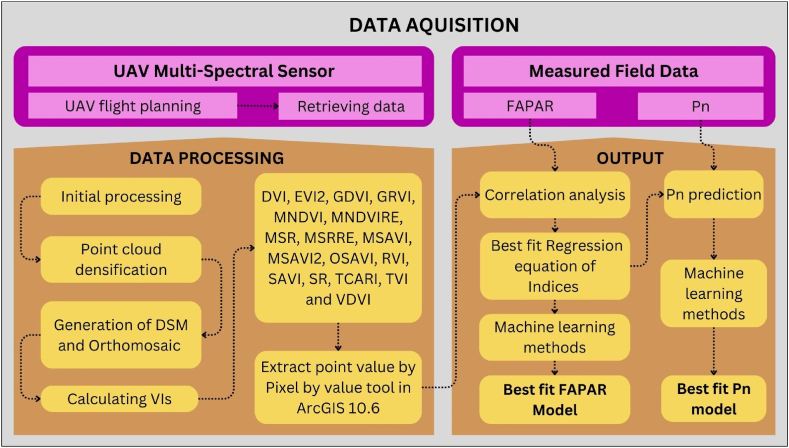
Flowchart of multispectral image processing.

The Pix4Dmapper software was employed to process (Fig. 3) the multispectral images gathered. After georeferencing, processing, and examination of the original data, an orthomosaic was generated. Furthermore, numerous overlapped images were compiled to produce an orthomosaic image with precise georeferencing. The reflectance values in each image of the multispectral data were normalized using the calibration panel images.

#### 2.3.2 Vegetation indices estimation

The primary tool for assessing aerial photographs was VIs. Initially, ArcGIS 10.6 software was employed to generate vegetation index maps from processed images. The processed data were then used to extract information, via*.,* VIs expressions. The vegetation index is crucial for assessing crop growth vigour, yield, and nutritional status. To differentiate maize from soil, pixels containing only maize and only soil were identified. Reflectance analysis at 720 nm showed distinct differences, with soil having lower reflectance. A 4 % reflectance threshold was used to classify pixels as vegetation (above 4 %) or soil (below 4 %). Pixels identified as maize were then used to calculate VIs using specified equations. VIs (*e.g.,* DVI, EVI2, GDVI, GRVI, MNDVI, MNDVIRE, MSR, MSRRE, MSAVI, MSAVI2, OSAVI, RVI, SAVI, SR, TCARI, TVI and VDVI) from Table 2 were valuable for predicting leaf area index, NDVI, and chlorophyll. Using ground data coordinates, the pixel-by-value tool in ArcGIS 10.6 was then employed to obtain various VIs from spectral data. Finally, statistical analysis was conducted on the retrieved VIs data.

**Table 2:** Vegetation indices were used in this study.

Category	Features	Expression	Applications	References
DVI	Difference Vegetation Index	$R_{840}-\;R_{664}$	Intercepted PAR	[35]
EVI2	Enhanced Vegetation Index 2	$2.5\times \frac{R_{840}-R_{668}\;}{1+R_{840}+2.4\times R_{668}}$	Intercepted PAR	[36]
GDVI	Green Difference Vegetation Index	$R_{840}-R_{560}$	Vegetation cover, LAI	[37]
GRVI	Green-Red Vegetation Index	$\frac{R_{560}-R_{668}}{R_{560}+R_{668}}$	Intercepted PAR	[38]
MNDVI	Modified Normalized Difference Vegetation Index	$\frac{R_{840}-R_{680}\;}{R_{840}+R_{680}-2\times R_{475}}$	Intercepted PAR	[39]
MNDVIRE	Modified Normalized Difference Vegetation Index Red-Edge	$\frac{R_{840}-R_{717}\;}{R_{840}+R_{717}-2\times R_{475}}$	Intercepted PAR	[40]
MSR	Modified Simple Ratio	$\frac{\frac{R_{840}}{R_{668}}-1}{\left(\sqrt{\frac{R_{840}}{R_{668}}}+1\right)}$	Intercepted PAR	[41]
MSRRE	Modified Simple Ratio Red-Edge	$\frac{\frac{R_{840}}{R_{717}}-1}{\left(\sqrt{\frac{R_{840}}{R_{717}}}+1\right)}$	Intercepted PAR	[42]
MSAVI	Modified Soil Adjusted Vegetation Index	0.5 × {(2$R_{840}$ +1 $){-\left(2R_{840}+1\right)}^{2}$ – 8 $\sqrt{R_{840}-R_{668}}$}	Intercepted PAR	[43]
MSAVI2	Modified Soil Adjusted Vegetation Index 2	0.5 × {(2$R_{840}$ +1)- $\sqrt{{\left(2R_{840}+1\right)}^{2}\;–\;8\;\left(R_{840}\;-\;R_{668}\right)}$}	Vegetation cover, LAI	[37]
OSAVI	Optimised Soil-Adjusted Vegetation Index	$\frac{R_{840}-R_{668}\;}{R_{840}+R_{668}+0.16}$	Intercepted PAR	[44]
RVI	Ratio Vegetation Index	$\frac{R_{668}\;}{R_{840}}$	Intercepted PAR	[45]
SAVI	Soil-Adjusted Vegetation Index	$\frac{1.5\times R_{840}-R_{668}\;}{R_{840}+R_{668}+0.5}$	Intercepted PAR	[46]
SR	Simple Ratio	$\frac{R_{840}}{R_{668}}$	Intercepted PAR	[47]
TCARI	Transformed Chlorophyll Absorption in Reflectance Index	3 $\times \{(R_{700}-R_{670}$) – 0.2 $(R_{700}-R_{550}$) $\times (\frac{R_{700}}{R_{670})})$}	Intercepted PAR	[14]
TVI	Ttransformed Vegetation Index	$\sqrt{\frac{R_{840}-R_{668}\;}{R_{840}+R_{668}}+0.5}$	Intercepted PAR	[48]
VDVI	Visible-Band Difference Vegetation Index	$\frac{2\times R_{560}-R_{668}-R_{475}}{2\times R_{560}+R_{668}+R_{475}}$	Intercepted PAR	[49]

### 2.4 Statistical analysis

The statistical validation of the results utilized R Studio, version 4.2.2 software. Initially, Pearson correlation analysis was employed to identify the most effective vegetation index strongly correlated with field-measured FAPAR data, determined by the correlation coefficient (r). Equation (2) outlines the calculation of the r-value, elucidating the correlation among the datasets. Model accuracy was evaluated through the computation of the coefficient of determination (R^2^) and root mean square error (RMSE) values. Regression analysis calculated R^2^ values for VIs as independent or predictor variables and field-measured values of FAPAR data as dependent or predicted variables. Increased predictability of the predictor variables from the predicted variable can be determined by higher R^2^ and lower RMSE values. Consequently, R^2^ and RMSE were determined using Equations (3), (4), respectively.

(2) $$r=\frac{n\sum P\;Q\;-\;\left(\sum P\right)\left(\sum Q\right)}{\sqrt{n\sum \left(P^{2}\right)\;-\;\left(\sum P^{2}\right)\;\sqrt{n\sum \left(Q^{2}\right)\;-\;\left(\sum Q^{2}\right)}}}\;\ldots$$

(3) $$R^{2}=\frac{\sum_{\;i=1}^{\;n}{\;\left(Q_{i}\;-{\;P}_{i}\right)}^{2}}{\sum_{\;i=1}^{\;n}{\;\left(Q_{i}\;-\;Q\right)}^{2}}\;\ldots$$

(4) $$\text{RMSE}=\sqrt{\frac{\sum_{\;i=1}^{\;n}{\;\left(P_{i}\;-{\;Q}_{i}\right)}^{2}}{n}}\;\ldots$$

In this case, P_i_ and Q_i_ stand for the estimated and measured values, respectively, and SD_Y_ for the standard deviation of the measured value. In the meantime, the average estimated and measured values were represented by the symbols P and Q, respectively. The sample number is denoted by the variable n.

### 2.5 Machine learning model algorithms

RF, XGB, SVM, and MLR models were used to establish non-linear relationships between selected indices and FAPAR and Pn values. Splitting data is an essential step in machine learning that ensures an accurate assessment of model performance. The dependent variables in this process are maize FAPAR and Pn, while the independent variables are multispectral vegetation indices. To begin this procedure, the dataset was split into two subsets: the training set, which contains 80 % of the dataset (100) to calibrate the model, and the testing set, which contains 20 % of the datasets (26) were applied to validate the model [50,51]. Data splitting guarantees that the models can produce accurate predictions on real-world data by discouraging overfitting (memorising the training set) and encouraging generalisation [51]. This procedure was repeated 100 times, and all VIs and those strongly correlated with FAPAR were utilized to develop the best-fit ML model for estimating maize canopy FAPAR. Predicted FAPAR values, derived from VIs, were used as predictor variables, while Pn was the predicted variable, various ML approaches were applied to identify the optimal model for forecasting Pn using scatter plots. R^2^ and RMSE values were used as indicators to evaluate the performance of the prediction models.

#### 2.5.1 Random forest (RF)

RF developed by Breiman [52], is an ensemble learning algorithm that combines the strength of decision trees with the bagging techniques [[53], [54], [55]]. Random Forest is recognized for its robustness in the presence of noisy data and its distinct advantage in curbing overfitting. A forest of decision trees is created, with each tree independently predicting outcomes using only a subset of the data. The final prediction was then determined by a majority vote among all the independent trees. The model was fitted using the randomForest package in R and was highly effective for addressing high-dimensional data in remote sensing and artificial intelligence applications. It can handle thousands of input variables without overfitting, making it ideal for feature selection. Additionally, Random Forest captures complex, non-linear relationships between features and the target variable, enhancing its utility in these fields [56,57].

#### 2.5.2 Extreme gradient boosting (XGBoost)

Chen and Guestrin's [58] Extreme Gradient Boosting (XGBoost) is an advanced and effective ensemble learning technique. By adding second derivatives via the Taylor expansion, it greatly improves upon conventional gradient boosting techniques and can better capture complex connections in data. Kearns and Valiant's [59] definitions of strong and weak learnable issues served as inspiration for XGBoost, which combines several weak learners through additive learning to produce a reliable and accurate prediction model. The XGBoost package in R was used to build this method, offering a full feature set that includes significant feature selection analysis based on metrics including gain, cover, and frequency. In general, XGBoost was quite good at managing intricate data patterns and obtaining excellent prediction performance in different machine learning [33].

#### 2.5.3 Support vector regression (SVR)

SVM include a variation called SVR that is intended for regression problems. It is particularly good at estimating continuous variables such as biophysical parameters and approximating functions [60]. The radial basis kernel function (RBF) was used for SVR in this work, and model tuning was used to identify the ideal values for gamma (γ) and the regularisation parameter (C). SVR is often used in remote sensing for both classification and regression tasks and is implemented using libraries such as Libsvm. It provides flexibility in selecting alternative kernel functions and fine-tuning parameters for the best possible model performance [21,57].

#### 2.5.4 Multiple linear regression (MLR)

Statistically, the connection between a dependent variable and many independent factors may be modelled using multiple linear regression (MLR). Predicting the dependent variable (such as FAPAR, Pn) using the weighted combination of independent variables (remote sensing characteristics) is made possible by the assumption that these variables have a linear relationship. Each independent variable is multiplied by the corresponding regression coefficient in the MLR equation to determine predictions, which were then summed with the intercept term. For the analysis of multivariate relationships, this method is popular because of its ease of use and interpretability [57].

## 3 Results

### 3.1 Measured value of various multi-spectral vegetation indices

**Fig. 4:**
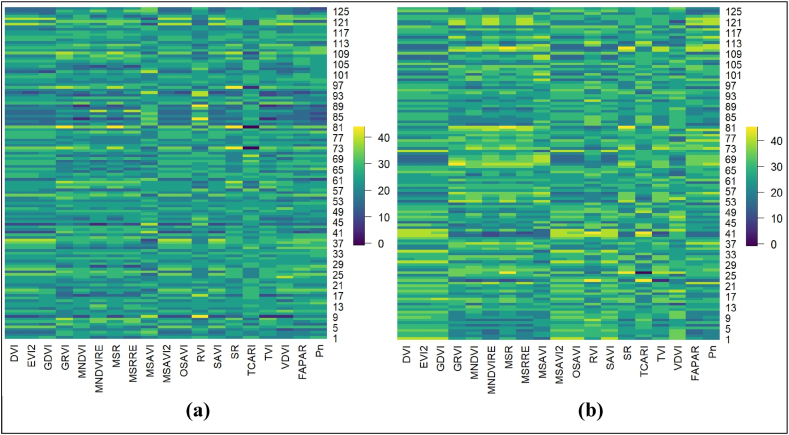
Heat map of different VIs and field-measured parameters (a. *kharif*; b. *rabi* season maize).

In this study different values of vegetation indices, *viz.*, DVI, EVI2, GDVI, GRVI, MNDVI, MNDVIRE, MSR, MSRRE, MSAVI, MSAVI2, OSAVI, RVI, SAVI, SR, TCARI, TVI and VDVI, and field-measured value of VIs, FAPAR, and Pn of maize were presented in Fig. 4.

**Fig. 5:**
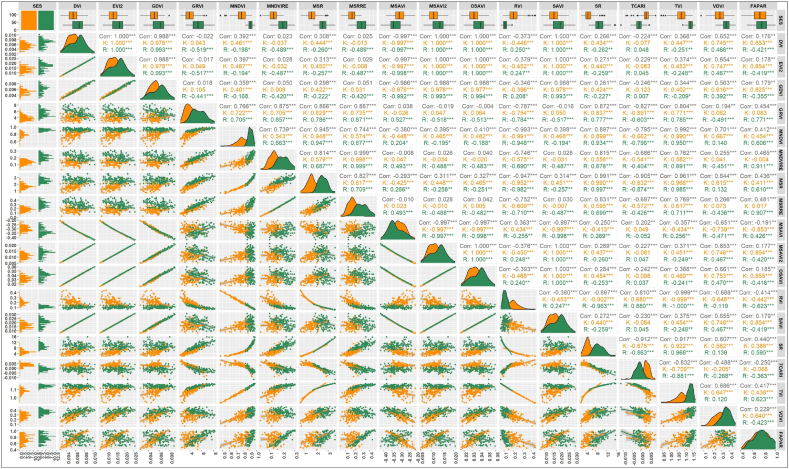
Correlation matrix for different vegetation indices and FAPAR. Note: Level of significance- ‘*’: p < 0.05, ‘**’: p < 0.01, ‘***’: p < 0.001, K: *kharif* and R: *rabi*.

Different VIs, *viz*., DVI from 0.003 to 0.009 and 0.003 to 0.010, EVI2 from 0.006 to 0.022 and 0.008 to 0.025, GDVI from 0.002 to 0.008 and 0.003 to 0.009, GRVI from 2.377 to 6.804 and 3.269 to 8.217, MNDVI from 0.467 to 0.928 and 0.836 to 0.975, MNDVIRE from 0.028 to 0.213 and 0.101 to 0.302, MSR from 0.706 to 2.962 and 1.736 to 3.579, MSRRE from 0.036 to 0.302 and 0.133 to 0.469, MSAVI from −0.387 to −205 and −0.420 to −0.227, MSAVI2 from 0.005 to 0.018 and 0.006 to 0.021, OSAVI from 0.016 to 0.051 and 0.019 to 0.059, RVI from 0.087 to 0.439 and 0.064 to 0.186, SAVI from 0.008 to 0.026 and 0.009 to 0.030, SR from 2.279 to 11.452 and 5.386 to 15.569, TCARI from −0.008 to 0.009 and −0.011 to 0.005, TVI from 0.943 to 1.157 and 1.089 to 1.174, VDVI from 0.069 to 0.455 and 0.233 to 0.466, FAPAR from 0.406 to 0.964 and 0.501 to 1.008, and Pn from 20.316 to 43.639 and 30.045–44.896 μmol. m^−2^. s^−1^, respectively. Fig. 4, Fig. 5 revealed that the VI values, FAPAR, and Pn readings differ from one another. This was due to differences at the field level, as the research field consisted of different treatments of indigenous technical knowledge-based formulations along with different times of applications for weed management in maize for experimental purposes.

### 3.2 Correlation analysis between indices and FAPAR

The Pearson correlation coefficient (r) for each VI and field-measured FAPAR data is illustrated in Fig. 4. Among the seventeen VIs, four during the *kharif* and nine in the *rabi* seasons exhibited negative correlations with field-measured FAPAR, with r values ranging from −0.853 to −0.004 during the *kharif* and −0.623 to −0.355 during the *rabi* season, respectively. The remaining VIs, six during the *kharif* and one in the *rabi* showed positive correlations with field-measured FAPAR data, although their r values were below 0.5. Seven VIs, namely DVI, EVI2, GDVI, MSAVI2, OSAVI, SAVI, and VDVI, demonstrated stronger positive correlations with field-measured FAPAR data during the *kharif* season, with r values of 0.853, 0.854, 0.825, 0.854, 0.855, 0.854 and 0.640, respectively. Similarly, during the *rabi* season, seven VIs including GRVI, MNDVI, MNDVIRE, MSR, MSRRE, SR, and TVI exhibited stronger positive correlations with field-measured FAPAR data, with r values, of 0.771, 0.606, 0.911, 0.610, 0.907, 0.595 and 0.623, respectively.

### 3.3 Linear regression analysis between indices and FAPAR value

The linear regression analysis was utilized using various VIs to predict FAPAR values during the *kharif* and *rabi* seasons. The results are summarized in Table 3, which includes regression equations, R^2^ values, and RMSE values for seventeen indices. The regression coefficients varied due to the diverse expressions of the indices. Among the seventeen VIs, ten showed a weak relationship with FAPAR, selected based on their R^2^ (<0.5) and RMSE (>0.1). However, seven VIs such as DVI, EVI2, GDVI, MSAVI, MSAVI2, OSAVI, and SAVI exhibited a stronger relationship, with R^2^ values ranging from 0.68 to 0.73 for predicting FAPAR. Notably, among all OSAVI had the highest R^2^ value of 0.73 and the lowest RMSE value of 0.70 during the *kharif* season. In contrast, during the *rabi* season, only two indices, MNDVIRE and MSRRE, demonstrated a very strong relationship, with R^2^ values of 0.830 and 0.823, and RMSE values of 0.055 and 0.057, respectively. Following them were GRVI, TVI, RVI, MSR, MNDVI, MSAVI, VDVI, DVI, MSAVI2, SAVI, EVI2, TCARI, and GDVI, which showed R^2^ value < 0.389 and RMSE value > 0.105, in predicting FAPAR.

**Table 3:** Regression equations of different VIs against FAPAR.

*Kharif* maize						
Sl. No.	VIs	Regression Equation	R^2^	Adj. R^2^	RMSE	Rank
1	DVI	y = 0.228 + 91.239x	0.728	0.726	0.071	5
2	EVI2	y = 0.225 + 37.117x	0.729	0.727	0.071	3
3	GDVI	y = 0.169 + 109.96x	0.681	0.679	0.077	7
4	GRVI	y = 0.674 + 0.013x	0.007	−0.001	0.135	14
5	MNDVI	y = 0.197 + 0.686x	0.207	0.200	0.121	9
6	MNDVIRE	y = 0.728 - 0.017x	0.00002	−0.008	0.135	17
7	MSR	y = 0.511 + 0.129x	0.169	0.162	0.124	12
8	MSRRE	y = 0.718 + 0.043x	0.0003	−0.008	0.135	16
9	MSAVI	y = −0.234 – 3.194x	0.727	0.725	0.071	6
10	MSAVI2	y = 0.228 + 45.78x	0.729	0.727	0.071	4
11	OSAVI	y = 0.210 + 15.941x	0.731	0.728	0.070	1
12	RVI	y = 0.913–0.894x	0.195	0.189	0.122	10
13	SAVI	y = 0.222 + 31.304x	0.729	0.727	0.071	2
14	SR	y = 0.562 + 0.031x	0.151	0.144	0.125	13
15	TCARI	y = 0.735–3.438x	0.004	−0.004	0.135	15
16	TVI	y = −0.817 + 1.433x	0.192	0.186	0.122	11
17	VDVI	y = 0.374 + 1.266x	0.409	0.404	0.104	8
*Rabi* maize						
1	DVI	y = 0.991–34.54x	0.177	0.170	0.122	11
2	EVI2	y = 0.992–14.03x	0.176	0.169	0.122	13
3	GDVI	y = 0.967–34.01x	0.126	0.119	0.126	17
4	GRVI	y = 0.254 + 0.093x	0.594	0.591	0.086	3
5	MNDVI	y = −1.561 + 2.56x	0.368	0.363	0.107	7
6	MNDVIRE	y = 0.208 + 2.706x	0.830	0.829	0.055	1
7	MSR	y = 0.197 + 0.221x	0.372	0.368	0.107	6
8	MSRRE	y = 0.275 + 1.634x	0.823	0.821	0.057	2
9	MSAVI	y = 1.194 + 1.307x	0.182	0.175	0.122	9
10	MSAVI2	y = 0.991–17.3x	0.176	0.170	0.122	12
11	OSAVI	y = 0.999–6.031x	0.173	0.166	0.122	15
12	RVI	y = 1.159 - 3.526x	0.388	0.383	0.105	5
13	SAVI	y = 0.993–11.85x	0.176	0.169	0.122	14
14	SR	y = 0.398 + 0.039x	0.354	0.349	0.108	8
15	TCARI	y = 0.745–20.89x	0.132	0.125	0.125	16
16	TVI	y = −4.927 + 4.993x	0.389	0.383	0.105	4
17	VDVI	y = 1.155–1.095x	0.179	0.172	0.122	10

*Note: y = dependent variable (FAPAR), x = dependent variables (VIs).

### 3.4 Prediction of FAPAR values of maize using machine learning methods

**Fig. 6:**
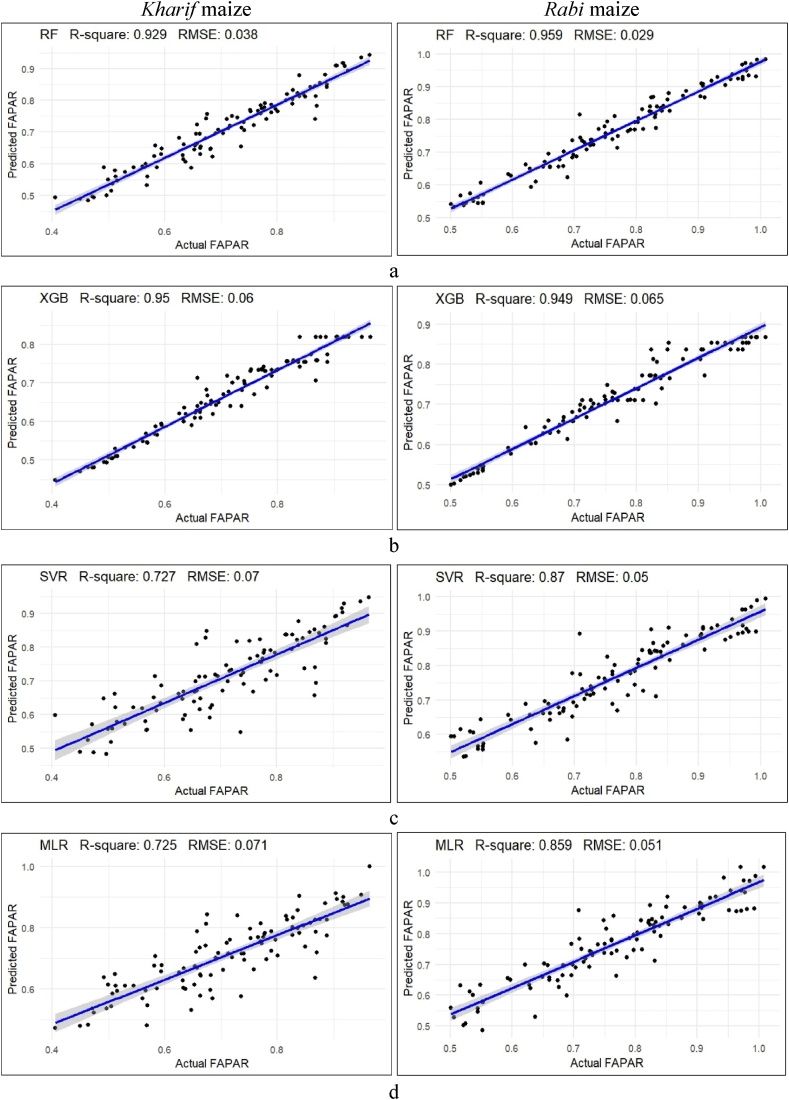
Different machine learning methods for prediction of FAPRA from all seventeen VIs during the *kharif* and *rabi* season (a. RF, b. XGBoost, c. SVR, d. MLR).

The evaluation of FAPAR using machine learning algorithms were performed with all VIs during the *kharif* and *rabi* season. It was evident from the analysis that RF consistently demonstrated superior performance in fitting the model by achieving the highest R^2^ values and the lowest RMSE values across both the *kharif* and *rabi* seasons, respectively, in Table 4 and Fig. 6. Specifically, during the *kharif* season, XGBoost exhibited an R^2^ value of 0.950 and an RMSE value of 0.060, followed by RF with an R^2^ value of 0.929 and an RMSE value of 0.038. In comparison, SVR, and MLR showed slightly lower R^2^ values of 0.727, and 0.725, and higher RMSE values of 0.070 and 0.071, respectively, indicating lower model fitting effect compared to XGBoost and RF. Nevertheless, SVR outperformed, with the highest R^2^ of 0.873 and the lowest RMSE of 0.045, indicating ideal predicted accuracy during the *kharif* season. Similarly, during the *rabi* season, RF continued to outperform other models with an impressive R^2^ value of 0.959 and an RMSE value of 0.029. In contrast, XGBoost, SVR, and MLR exhibited R^2^ values of 0.949, 0.870, and 0.859, respectively, and RMSE values of 0.065, 0.05, and 0.051, respectively, for fine-tuning the model. The SVR model demonstrated significant fitting accuracy throughout the *kharif* season, with the highest R^2^ and the lowest RMSE values of 0.873 and 0.045, respectively, for fine-tuning the model. Nevertheless, MLR outperformed during the *rabi* season, with the highest R^2^ and the lowest RMSE values of 0.838 and 0.053, respectively, indicating ideal fitting accuracy for FAPAR estimation with all seventeen VIs.

**Table 4:** Prediction accuracy of different model against FAPAR and Pn.

Model	*Kharif* maize		*Rabi* maize	
	R^2^	RMSE	R^2^	RMSE
Prediction of FAPAR with all the VIs				
RF	0.857	0.046	0.818	0.059
XGBoost	0.719	0.089	0.796	0.089
SVR	0.873	0.045	0.802	0.065
MLR	0.839	0.050	0.838	0.053
Prediction of FAPAR with seven VIs (r > 0.55)				
RF	0.852	0.047	0.810	0.059
XGBoost	0.657	0.093	0.794	0.086
SVR	0.844	0.049	0.820	0.054
MLR	0.873	0.044	0.822	0.054
Prediction of Pn				
RF	0.672	2.858	0.961	0.854
XGBoost	0.701	7.061	0.920	6.650
SVR	0.721	2.689	0.963	0.838
MLR	0.741	2.531	0.955	1.070

**Fig. 7:**
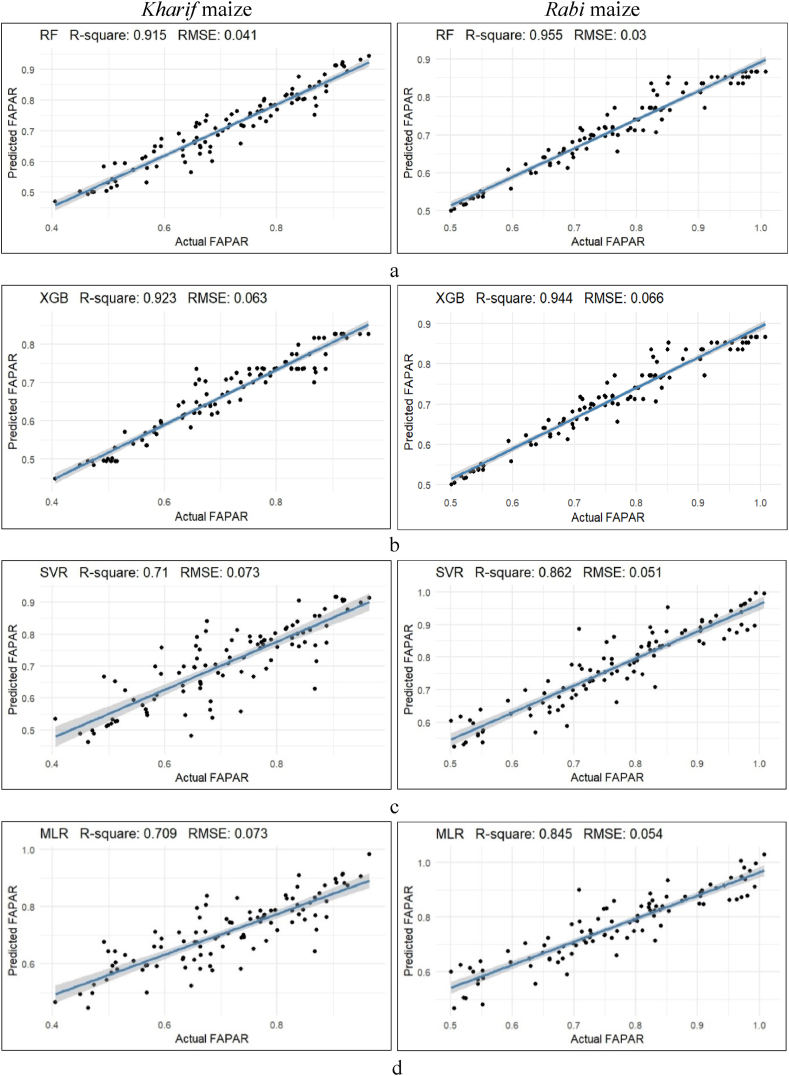
Different machine learning methods for prediction of FAPRA from seven VIs (DVI, EVI2, GDVI, MSAVI2, OSAVI, SAVI and VDVI) during the *kharif* and *rabi* season (a. RF, b. XGBoost, c. SVR, d. MLR).

In the preceding scenario, all the VIs were used to predict FAPAR, another scenario where seven predictor variables were selected based on their correlation coefficient values, r > 0.5 with FAPAR. These include DVI, EVI2, GDVI, MSAVI2, OSAVI, SAVI, and VDVI, as well as GRVI, MNDVI, MNDVIRE, MSR, MSRRE, SR, and TVI from both the *kharif* and *rabi* seasons, respectively, used for the prediction of FAPAR for best fitting effect and accuracy rate is shown in Fig. 7 and Table 4.

RF and XGBoost persistently showed better fine-tuning the model with higher R^2^ values of 0.923 and 0.915, and lower RMSE values of 0.063 and 0.041, than SVR and MLR, with R^2^ values of 0.710 and 0.709, and RMSE values of 0.073 each, respectively, during the *kharif* season. Conversely, during the *rabi* season, RF with an R^2^ value of 0.955 and RMSE value of 0.030, showed greater fitting of the model than, XGBoost, SVR, and MLR with values of R^2^ and RMSE were 0.944 and 0.051, 0.862 and 0.054, and 0.845 and 0.066, respectively. During the *kharif* season, the MLR model demonstrated great fitting accuracy with a higher R^2^ value of 0.873 and a lower RMSE value of 0.044. During the *rabi* season, MLR and SVR both outperformed one another in terms of predicted accuracy, recording the same R^2^ and RMSE values of 0.82 and 0.054, respectively.

These findings underscore the effectiveness of RF in predicting the best-fitting effect of FAPAR values across different seasons. SVR of all VIs and MLR of the seven superior VIs models showed significant accuracy, both achieving a fitting accuracy of 87 % during the *kharif* season. However, during the *rabi* season, MLR varied between 82 % and 84 % accuracy in both scenarios. This indicated a consistent accuracy rate in both scenarios.

### 3.5 Prediction of Pn values of maize using machine learning methods

**Fig. 8:**
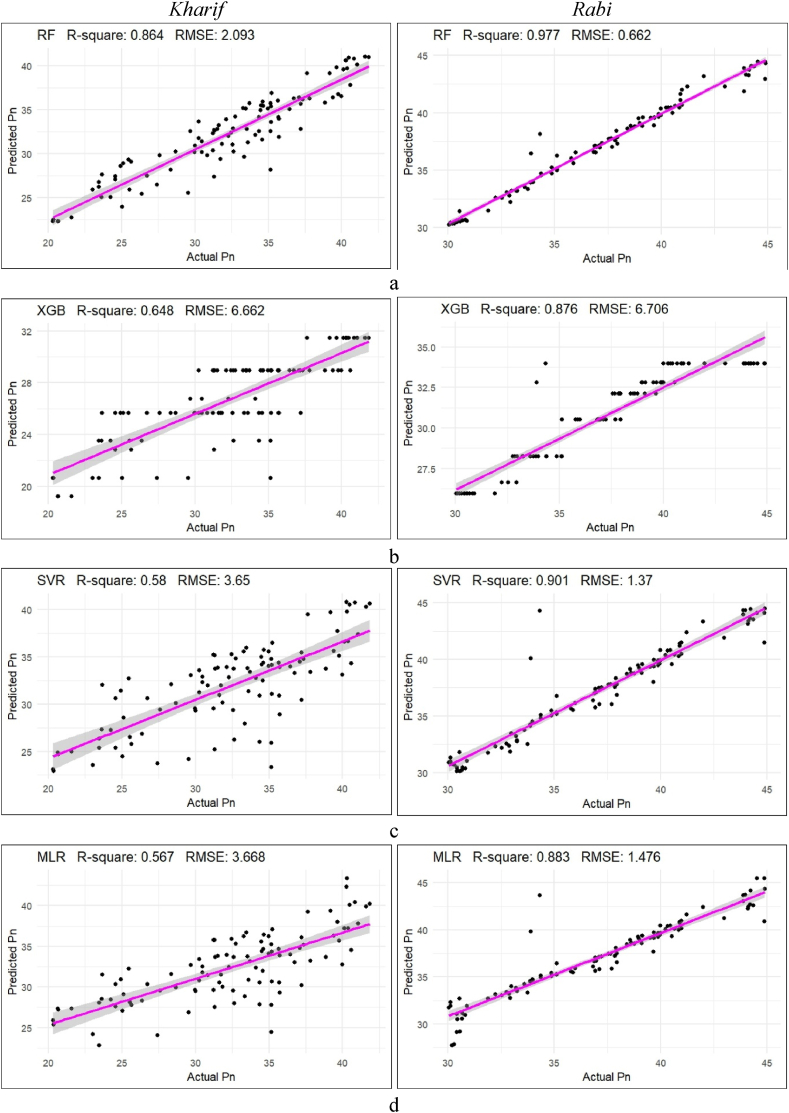
Different machine learning methods for prediction of Pn from seven VIs during the *kharif* (OSAVI) and *rab*i (MNDVIRE) season (a. RF,b. XGBoost, c. SVR, d. MLR).

Among all the vegetation indices, OSAVI during the *kharif* season and MNDVIRE during the *rabi* season stood out with the highest R^2^ values of 0.731 and 0.830, and the lowest RMSE values of 0.055 and 0.070, respectively. As the Fraction of absorbed photosynthetically absorbed radiation is directly proportional to the photosynthesis rate of a plant, these indices’ predicted values with FAPAR were then utilized to predict the net photosynthesis rate (Pn), which was subsequently subjected to different machine learning algorithms to determine the best fit with the highest accuracy, shown in Fig. 8 and Table 4.

In the prediction of photosynthesis rate, RF exhibited the best fit by achieving the highest R^2^ value of 0.864 and the lowest RMSE value of 2.093, followed by XGBoost, SVM, and MLR with R^2^ and RMSE values of 0.648 and 6.662, 0.580 and 3.650, and 0.567 and 3.668, respectively. Among these models, MLR demonstrated better accuracy performance with a higher R^2^ and lower RMSE value of 0.741 and 2.531, respectively, followed by SVM, XGBoost, and RF with R^2^ values of 0.721, 0.701, and 0.672, and RMSE values of 2.689, 7.061, and 2.858, respectively, during the *kharif* season. Similarly, during the *rabi* season, the trends remained consistent as RF achieved the best fitting effect with the highest R^2^ value of 0.977 and the lowest RMSE value of 0.662 among all the ML models. SVR performed better predicting accuracy with a higher R^2^ value of 0.963 and lower RMSE value of 0.838 than RF, MLR, and Boost, for the prediction of Pn values during the *rabi* season.

These findings suggest that machine learning algorithms, particularly RF, were effective in fitting photosynthesis rates. MLR and SVR showed promising accuracy rates of the model, especially in specific seasonal contexts.

## 4 Discussion

### 4.1 Prediction of FAPAR and Pn with VIs

Accurate estimates of canopy property have new opportunities owing to multispectral remote sensing. OSAVI, SAVI, EVI2, MSAVI2, DVI, and GDVI among all the indices showed a strong correlation with the FAPAR prediction during the *kharif* season. When it comes to precisely measuring FAPAR, EVI2 performs better than SR, NDVI, DVI and GDVI. The reason for EVI2's higher performance was that it incorporates input reflectance that has been partly or atmospherically adjusted for ozone absorption and Rayleigh scattering [61,62]. Furthermore, EVI2 is insensitive to the majority of canopy backgrounds due to its canopy background adjustment factor (2.5), which reduces the effects of non-linear, differential NIR, and red radiant transmission inside the canopy [61,62]. Unlike other VI, which tends to saturate and be impacted by the background in high LAI circumstances, this insensitivity enables EVI2 to display great sensitivity to FAPAR even at high Leaf Area Index (LAI) values and decrease noise due to background and atmospheric interferences [63,64]. OSAVI is distinguished by its unique technical advantages over SAVI and MSAVI 2. While SAVI and MSAVI 2 default to a soil-adjustment factor of 0.5, OSAVI's development included an optimised coefficient of 0.16, which was strategically chosen to lessen the NDVI's susceptibility to variations in the soil background across a range of environmental scenarios. Because of this optimised adjustment and its hybrid design, which combines components from orthogonal (PVI: Perpendicular Vegetation Index) and ratio-based (NDVI, SAVI) indices, OSAVI is better able to measure plant cover correctly while reducing soil interference [2,65,66].

However, during the *rabi* season, MNDVIRE and MSRRE demonstrated a very strong relationship with measured FAPAR, compared with other vegetation indices and these indices have a red-edge band which makes the differentiation with other indices. Red-edge NDVI was the most effective index for overcoming NDVI's reduced sensitivity to FAPAR at higher biomass densities [[67], [68], [69]]. The red-edge index proved the most accurate for FAPAR estimation during the *rabi* season. This is because the red-edge position's spectral characteristics are crucial for describing green vegetation information within reflectance spectra in the 680–750 nm range [6,70,71]. The red-edge index strong correlation with chlorophyll concentration, making it a valuable indicator for estimating chlorophyll content, biomass, LAI, and other environmental factors [[72], [73], [74]]. Studies have consistently shown REP's efficacy, making it a preferred choice for monitoring vegetation dynamics and health. The challenges in accurately estimating FAPAR, including saturation issues with traditional VIs, limitations beyond certain thresholds of FAPAR, LAI (>2 or 3), biomass values (0.3 g cm^−2^), and the impact of phenological stages on spectral reflectance [[75], [76], [77]]. To address these challenges, they emphasized the significance of instruments like the multispectral sensor incorporating specialized red-edge bands for enhanced monitoring capabilities of red-edge region's sensitivity to canopy variations making it superior to other indices, addressing saturation issues and providing accurate estimates even in challenging conditions.

### 4.2 Comparison of different machine learning models

In predicting FAPAR and Pn, RF outperformed XGBoost, SVR and MLR in terms of model fitting, as evidenced by significantly higher R^2^ and lower RMSE values. RF's advantages include mitigating overfitting through the introduction of randomness, facilitating easy parallelization, handling high-dimensional data without the need for dimension reduction, and maintaining relatively high accuracy even with a limited number of datasets [20,78]. In comparison to all ML models, SVR and MLR, performed better in estimating FAPAR with high fitting accuracy during the *kharif* and *rabi*, respectively. However, XGBoost demonstrated the lowest fitting accuracy across all seasons. The SVR and MLR were better suited for small sample sizes and might become unpredictable when modelling huge datasets, which would reduce their capacity to make predictions and represent the sample's underlying patterns [2,43]. When predicting FAPAR based on seven indices variables, all models demonstrate a high degree of prediction accuracy, with a mean average accuracy of 81 % during the *rabi* season. Out of four machine learning techniques, XGBoost performed inadequately during the *kharif* season, with a mid-accuracy performance of 65.70 % accuracy. This might be because it's difficult to identify significant patterns in the data after removing the 10 vegetation indices, or it could be because the dataset has a lot of noise and outliers [[30], [31], [32], [33],^58^].

Prediction of net photosynthetic rate using a single variable, random forests had a lower fitting accuracy of 67 %, whereas multiple linear regression recorded a better fitting accuracy of 74 %. Since decision trees are used for high-dimensional data training and the RF model relies on huge samples with tens of thousands or more occurrences, training RF models with small sample sizes is troublesome [57,79]. Therefore, the medium data size (100–1000) in our experiments could have played a role in the poor performance of the RF models. The MLR approach may be used to represent the linear relation between variables when there is a clear linear relationship between the dependent and independent variables. Nevertheless, it is possible to ignore the linear relationship that exists between the dependent and independent variables, which makes using MLR to estimate easy [80]. All models for prediction of Pn with single variables during the *rabi* season demonstrated superior prediction accuracy with more than 90 %, and SVR achieved the highest accuracy rate of 96.3 %, followed by RF. It is not always possible to increase the prediction accuracy by combining more dependent variables, such as vegetation indices [33]. Random Forest is useful in minimising overfitting and managing missing values but SVR was better suited for small sample sizes. When simulating large amounts of data, SVR may become unpredictable and lose its ability to predict outcomes and reflect the underlying patterns of the sample [81,82]. SVR significantly improved R^2^ and RMSE compared to RF. As a novel small-sample learning method with a strong theoretical foundation, SVM avoids traditional inductive-deductive processes, enabling efficient “transduction reasoning” from training to prediction samples, thereby simplifying classification and regression tasks [83]. SVM demonstrated good robustness since adding or removing non-support vector samples did not affect the model [81]. It performed well with limited data, making it valuable when sample numbers were scarce.

## 5 Novelty

Several studies have estimated maize FAPAR using UAV-based multispectral and hyperspectral data [2,63,71]. However, this study represents the first attempt to explore the potential of utilizing UAV-based multispectral data to estimate maize Pn from FAPAR in a combined manner. The results of this study demonstrate a moderate improvement in estimating maize photosynthetic characteristics, which may be attributed to the direct proportionality between maize canopy FAPAR and Pn. Additionally, the vegetation indices (VIs) considered in this study effectively represent FAPAR information. The findings confirm that understanding the biochemical and biophysical characteristics of plants can significantly aid in monitoring growth and yield, thereby supporting sustainable crop management practices and benefiting farmers and policymakers alike. This research underscores the novel integration of UAV-based multispectral data with photosynthetic characteristic estimation, providing a valuable contribution to precision agriculture.

## 6 Limitations

Estimating maize FAPAR at different times during the growing season can more accurately monitor maize growth and estimate yield. This study estimated maize FAPAR using multispectral data from only one time period due to experimental and cost limitations, focusing on the critical stage. However, the methods demonstrated are effective and can be applied to estimate maize FAPAR throughout the entire growing season when comprehensive datasets are available.

The main challenges in maize photosynthetic characteristics prediction from unrepresentative training datasets and the inability of machine learning approaches to utilize all useful information. To address this, datasets should integrate RGB, multispectral, and hyperspectral sensors to enhance predictive accuracy. Additionally, conventional machine learning models such as RF, XGB, SVR, and MLR used in this study could be improved by testing and integrating advanced deep learning techniques with new feature selection methods to evaluate more advanced models for predicting FAPAR and Pn values in future studies.

## 7 Conclusion

The study aimed to analyse the potential of using UAV-based multispectral imaging and machine learning algorithms to predict FAPAR and Pn during the *kharif* and *rabi* seasons of maize. Seventeen vegetation indices derived from UAV multispectral data, along with two field-measured FAPAR and Pn data points, were used in four ML models. The study found that several vegetation indices, such as OSAVI, SAVI, EVI2, MSAVI2, during the kharif and MNDVIRE, and MSRRE, during the rabi season showed strong relationships with higher R^2^ values and lower RMSE values. Among the four ML models (RF, XGBoost, SVR and MLR) tested, RF demonstrated the best overall performance in predicting FAPAR, especially when considering all variables or only the strongly correlated seven variables. However, MLR and SVR showed better fitting accuracy for FAPAR prediction. Similarly, RF showed the best fitting effect for predicting net photosynthesis rate, with high R^2^ values of 0.864 and 0.977 while MLR and SVR achieved higher accuracy but still notable results of 74.1 % and 96.3 %, during the *kharif* and *rabi* seasons, respectively. The results indicated that RF excelled in fine-tuning the model, while SVM and MLR were more effective in achieving higher accuracy with superior R^2^ and RMSE values. Consequently, it is strongly recommended to adopt an optimal combination of various spectral indices for estimating the photosynthetic characteristics of maize. The study emphasizes the significant potential of ML methods in accurately estimating these characteristics across different seasons, providing valuable insights for biomass prediction, vegetation productivity modelling, and yield estimation.

## Funding

This research did not receive any specific funding.

## Data availability statement

Data of this study has not been associated or deposited into a publicly available repository. All relevant data are included in the article. Data will be made available on request.

## CRediT authorship contribution statement

**Pradosh Kumar Parida:** Writing – review & editing, Writing – original draft, Visualization, Validation, Supervision, Software, Resources, Methodology, Investigation, Formal analysis, Data curation, Conceptualization. **Somasundaram Eagan:** Writing – review & editing, Visualization, Validation, Supervision, Resources, Project administration, Methodology, Investigation, Formal analysis, Data curation, Conceptualization. **Krishnan Ramanujam:** Writing – review & editing, Visualization, Validation, Supervision, Methodology, Conceptualization. **Radhamani Sengodan:** Writing – review & editing, Visualization, Validation, Supervision, Methodology, Conceptualization. **Sivakumar Uthandi:** Writing – review & editing, Visualization, Validation, Supervision. **Parameswari Ettiyagounder:** Writing – review & editing, Visualization, Validation, Supervision, Conceptualization. **Raja Rajagounder:** Writing – review & editing, Visualization, Validation, Supervision, Methodology, Conceptualization.

## Declaration of competing interest

The authors declare no conflict of interest related to this article.
